# Comparison of arthroscopic suture-bridge technique and double-row technique for treating rotator cuff tears: A PRISMA meta-analysis

**DOI:** 10.1097/MD.0000000000015640

**Published:** 2019-05-17

**Authors:** Yi-Ming Ren, Hong-Bin Zhang, Yuan-Hui Duan, Yun-Bo Sun, Tao Yang, Meng-Qiang Tian

**Affiliations:** a Department of Orthopedics, People's Hospital of Jinxiang County, Jinxiang, Shandong Province; b Department of Joint and Sport Medicine, Tianjin Union Medical Center, PR China.

**Keywords:** double-row, meta-analysis, rotator cuff tear, suture-bridge, systematic review

## Abstract

**Background:**

Rotator cuff tear is a common shoulder disorder in the elderly. Either arthroscopic double-row (DR) or suture-bridge (SB) technique for rotator cuff tear patients is needed to choose. We conducted this systematic review and meta-analysis to compare the clinical outcomes of arthroscopic SB versus DR intervention.

**Methods:**

The 7 studies were acquired from PubMed, Medline, Embase, CNKI, Google, and Cochrane Library. The data were extracted by 2 of the co-authors independently and were analyzed by RevMan5.3. Mean differences (MDs), odds ratios (ORs), and 95% confidence intervals (CIs) were calculated. Cochrane Collaboration's Risk of Bias Tool and Newcastle–Ottawa scale were used to assess risk of bias.

**Results:**

Seven studies including 1 randomized controlled trial and 6 observational studies were assessed. The methodological quality of the trials ranged from low to moderate. The pooled results of American Shoulder and Elbow Surgeons score, Constant score, visual analog scale score, and range of motion showed that the differences were not statistically significant between the 2 interventions. The difference of University of California at Los Angeles (UCLA) score was statistically significant between SB and DR intervention, and SB treatment was more effective (MD = −0.95, 95% CI = −1.70 to −0.20, *P* = .01). The difference of re-tear rate was statistically significant and SB treatment achieved better result than DR treatment (OR = 0.31, 95% CI = 0.15–0.64, *P* = .001). Sensitivity analysis proved the stability of the pooled results and the publication bias was not apparent.

**Conclusions:**

Both arthroscopic SB and DR interventions had benefits in rotator cuff tear. SB treatment was more effective in UCLA score and had lower re-tear rate than DR treatment. The arthroscopic SB technique is recommended as the optical choice for rotator cuff tear.

## 1 Introduction

The rotator cuff tear is a common cause of shoulder pain and dysfunction. The incidence of this disease increases with age, more than 50% over 70 years of age.^[1,2]^ Rotator cuff tear is usually characterized by impact pain, nocturnal pain, and shoulder joint dysfunction, which seriously affect the life and working ability of patients, and reduce the quality of life of patients. The treatment of rotator cuff tear includes surgical treatment and nonsurgical treatment. The patients with the duration of this disease are less than 3 months, the tearing degree is lighter, the age is older and the body is poor can be treated by conservative treatment.^[3]^

Arthroscopic rotator cuff repair is known to be a successful procedure that restores function and provides satisfactory pain relief when nonoperative treatment has failed. Arthroscopic surgery has the advantages of minimal invasion, quick postoperative recovery and high acceptance. The methods of repairing rotator cuff tear under arthroscopy are single-row anchor fixing technique, traditional double-row (DR) anchor fixing technique and suture-bridge (SB) technique.^[4]^ The traditional DR technology has advantages over the single-row fixation in the coverage and initial fixation strength of the rotator cuff. However, more anchors are used to reduce the healing area, and there are more knots on the surface of the rotator cuff, which are easy to form adhesion or cause new impingement.^[5,6]^ Biomechanical studies have shown that the recently introduced arthroscopic SB technique improved the pressurized contact area and mean pressure between the tendon and footprint compared with conventional DR techniques. In addition, SB repair may allow quick arthroscopic cuff repair with reduced knot impingement compared with conventional DR techniques.^[7–9]^

Up to now, some clinical studies compared structural and functional outcomes between SB and conventional DR techniques. However, there have been no systematic, quantitative evaluations between 2 techniques. In this article, we included 7 relevant studies to compare the clinical outcomes of SB and conventional DR techniques in rotator cuff tear to provide some evidence for clinical decision making.

## 2 Materials and methods

Ethical approval or patient consent was not required since the present study was a review of previously published literatures.

### 2.1 Inclusive criteria of published studies

#### 2.1.1 Types of studies

We considered all published and unpublished studies covering randomized controlled trials (RCTs), and observational studies including retrospective and prospective studies.

#### 2.1.2 Types of participants

All patients had been diagnosed as rotator cuff tears, regardless of the diagnostic criteria used, etiology of the disease, associated pathology, gender, and age.

#### 2.1.3 Types of interventions

All surgical techniques including the arthroscopic SB technique or DR suture-bridging and the arthroscopic conventional DR technique, were considered. The exclusion criteria were as follows:

(1) insufficient clinical outcome data in studies and
(2) reviews, letters, or conference articles.

#### 2.1.4 Types of outcome measures

The primary outcome measures were the clinical outcomes synthesizing the American Shoulder and Elbow Surgeons (ASES) score, Constant score, the Shoulder Rating Scale of the University of California at Los Angeles (UCLA) score and the visual analog scale (VAS) score. The secondary outcomes included:

(1) postoperative active range of motion (ROM) (forward flexion, external rotation), and
(2) re-tear rate.

#### 2.1.5 Search methods for identification of studies

Six databases (PubMed, Medline, Embase, CNKI, Google, and Cochrane Library) were searched using the keywords such as “rotator cuff tear or rotator cuff injuries or rotator cuff tear arthropathy,” “double-row or double row,” “suture-bridge or suture-bridging,” “surgery or surgical or operation,” and “arthroscopic or arthroscopy” through January 2018 to collect relevant studies about the clinical comparisons of SB versus conventional DR intervention in rotator cuff tears. The titles and abstracts of potential related articles identified by the electronic search were reviewed. References from retrieved articles were also assessed to extend the search strategy.

#### 2.1.6 Data collection and quality assessment

Two partners (TY, WJZ) independently assessed the titles and abstracts of all the studies screened during initial search, and they excluded any clearly irrelevant studies using the inclusion criteria. Data were independently extracted using a standard data form for the first author's name, year of publication, sample size, gender, age, intervention, country, study design, follow-up, and relevant outcomes. A third partner (YHD) would handle any disagreement about inclusion of a study and reach a consensus. Cochrane Collaboration's Risk of Bias Tool was manipulated for the appraisal of RCT study quality. Observational studies were assessed by the Newcastle–Ottawa scale including 8 items. A higher overall score indicates a lower risk of bias and a score of 5 or less (out of 9) corresponds to a high risk of bias.

#### 2.1.7 Statistical analysis

RevMan statistical software5.3 was used for meta-analysis. The continuous variables would be conducted by mean difference (MD) and 95% confidence interval (CI). For the dichotomous outcome, we calculated the odds ratios (ORs) and 95% CIs. The chi-squared statistic and the *I*^2^ statistic were used for the test of heterogeneity. A *P* < .05, *I*^2^ > 50% was considered a significant heterogeneity, and random-effect models were applied. Otherwise fixed-effect models were used if there was no significant heterogeneity (*P* ≥ .05, *I*^2^ ≤ 50%). We also performed sensitivity analysis by omitting 1 study at a time to test the stability of the pooled results. Publication bias was shown by the funnel plot.

## 3 Results

### 3.1 Studies identification and inclusion

**Figure 1:**
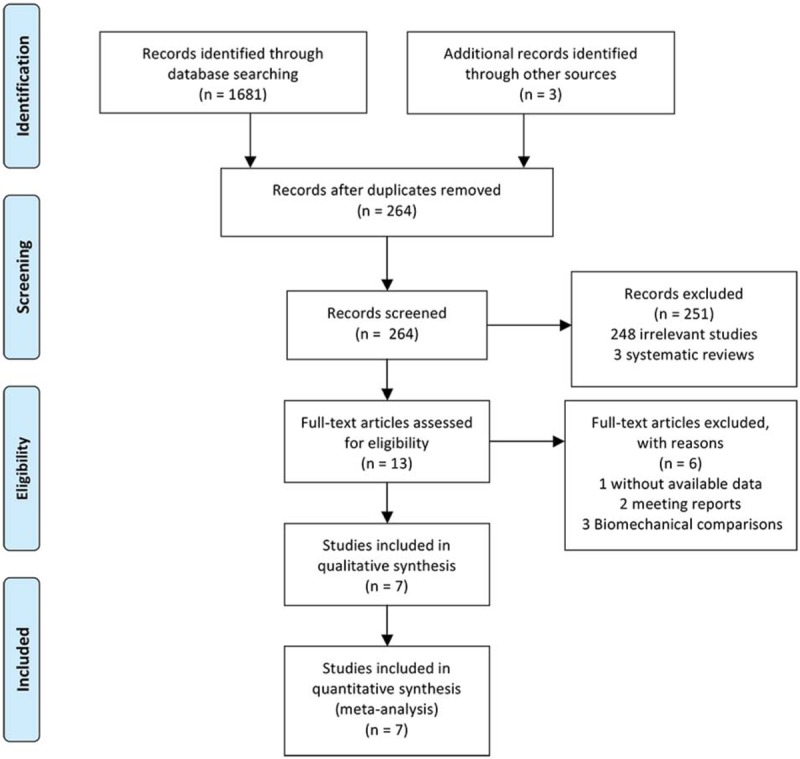
PRISMA flow diagram. PRISMA = preferred reporting items for systematic reviews and meta-analyses.

Searches conducted in the PubMed, Medline, Embase, CNKI, Google, Cochrane Library databases, and other sources, yielded a total of 1684 articles. After removing duplicates, 264 literatures were remained. Based on the titles and abstracts review, 248 irrelevant articles, and 3 systematic reviews of them were excluded. Thirteen full-text articles were assessed for eligibility. However, 6 articles were excluded based on the previously established exclusion criteria (1 without available data, 2 meeting reports, and 3 biomechanical comparisons). Finally, 7 trials (1 RCT and 6 observational studies) were included in this systematic review and meta-analysis. The detail of selection process is listed in Figure 1.

### 3.2 Study characteristics

We assessed 7 studies^[10–16]^ including 1 RCT, 3 retrospective studies, and 3 comparative cohort studies in this article. The included studies were conducted in 3 countries (Japan, Korea, and China) from 2011 to 2017, and involved 585 patients (290 patients treated with SB technique, 295 patients treated with DR technique) aged 49.2 to 63.9 years. The average follow-up duration ranged from 6 to 62 months. The clinical outcomes of the studies were evaluated mainly based on ASES score, Constant score, UCLA score, VAS score, Japanese Orthopaedic Society score, ROM, and re-tear rate. The detailed information of included studies is shown in Table 1.

**Table 1:**
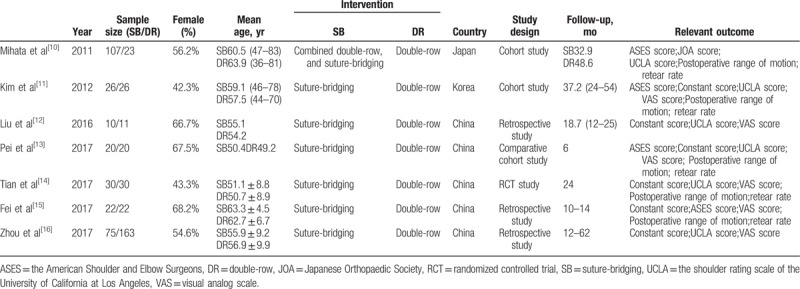
Characteristics of studies included.

### 3.3 Methodological assessment of study quality

**Figure 2:**
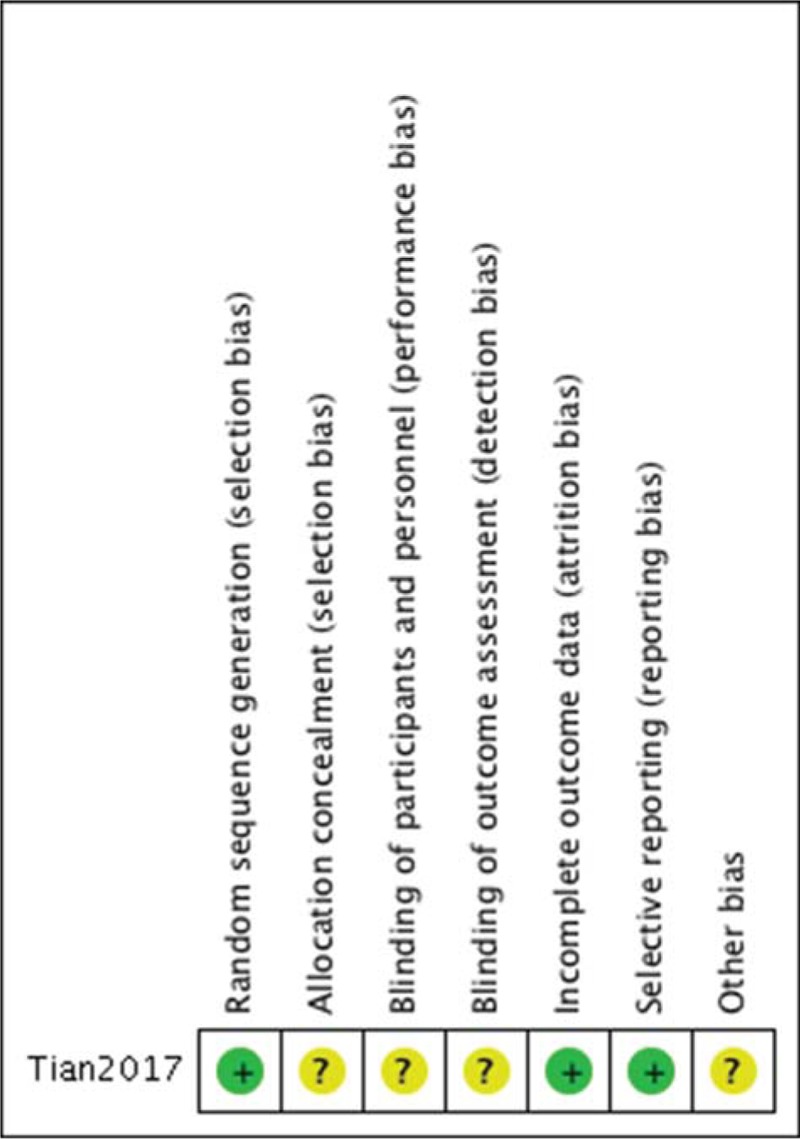
Risk of bias summary: this risk of bias tool incorporates the assessment of randomization (sequence generation and allocation concealment), blinding (participants and outcome assessors), incomplete outcome data, selective outcome reporting, and other risk of bias. The items were judged as “low risk,” “unclear risk,” or “high risk.” Green means “low risk,” red means “high risk,” and yellow means “unclear risk.”

Methodological quality assessment of the 7 included studies is presented in Figure 2 and Table 2. Among the RCT, Tian's study^[14]^ clearly described the random sequence generation by random number tables, but the blinding and allocation concealment were not mentioned, which could be regarded as a low quality study. Among the observational studies, the Newcastle–Ottawa scale including the exposed cohort, the nonexposed cohort, ascertainment of exposure, outcome of interest, comparability, assessment of outcome, length of follow-up, and adequacy of follow-up, was used to assess the risk of bias. The scores of all 6 studies ranged from 7 to 8, indicating a low risk of bias.

**Table 2:**
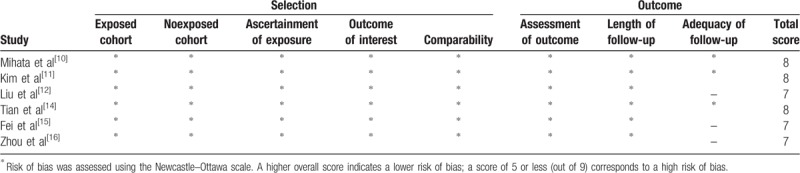
Risk of bias was assessed using the Newcastle-Ottawa Scale.

### 3.4 Comparison of constant score between SB and DR

**Figure 3:**
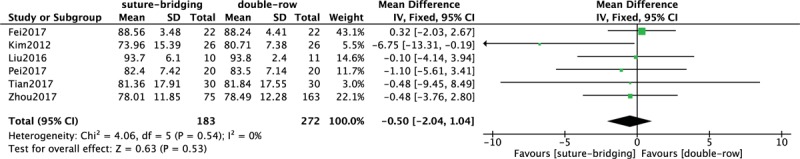
Forest plot of comparison: Constant score between traditionalDR technique and SB technique. DR = double-row, SB = suture-bridge.

Comparison of postoperative constant score between SB and DR was conducted among the 6 included studies,^[11–16]^ which included 455 patients (183 patients receiving SB and 272 patients receiving DR), as shown in Figure 3. Heterogeneity testing showed that there was no heterogeneity among the studies (*P* = .54, *I*^2^ = 0%), so the fixed-effect model was used to pool the data from the 6 studies. The pooled result showed that the difference was not statistically significant between the SB group and the DR group (MD = −0.50, 95% CI = −2.04 to −1.04, *P* = .53).

### 3.5 Comparison of ASES score between SB and DR

**Figure 4:**
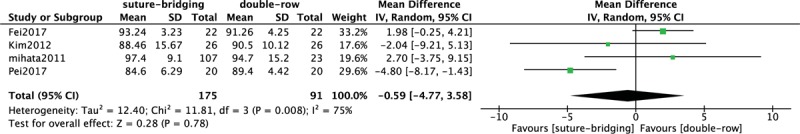
Forest plot of comparison: ASES score between traditional DR technique and SB technique. ASES = American Shoulder and Elbow Surgeons, DR = double-row, SB = suture-bridge.

Comparison of postoperative ASES score between SB and DR was conducted between the 4 included studies,^[10,11,13,15]^ which enrolled 266 patients (175 patients receiving SB and 91 patients receiving DR), as shown in Figure 4. Heterogeneity testing showed that there was moderate heterogeneity between the studies (*P* = .78, *I*^2^ = 75%), so the random-effect model was used to pool the data for the 2 groups. The overall estimate showed that the difference was not statistically significant between the SB group and the DR group (MD = −0.59, 95% CI = −4.77–3.58, *P* = .78).

### 3.6 Comparison of VAS score between SB and DR

**Figure 5:**
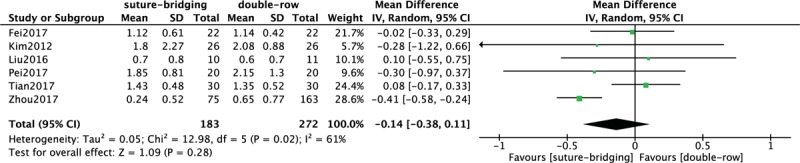
Forest plot of comparison: VAS score between traditional DR technique and SB technique. DR = double-row, SB = suture-bridge, VAS = visual analog scale.

Comparison of postoperative VAS score between SB and DR treatment was conducted among 6 included studies^[11–16]^ which contain 455 patients in Figure 5. A heterogeneity test showed that there was the moderate heterogeneity among studies (*P* = 0.02, *I*^2^ = 61%), so the random-effect model was used. The overall estimate showed that the difference between the 2 groups was not statistically significant (MD = −0.14, 95% CI = −0.38–0.11, *P* = .28).

### 3.7 Comparison of UCLA score between SB and DR

**Figure 6:**
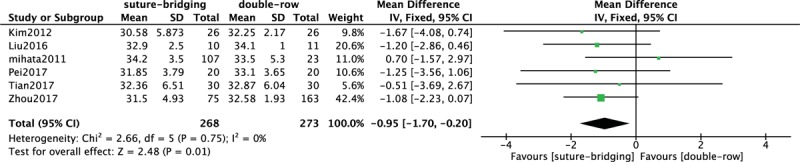
Forest plot of comparison: UCLA score between traditional DR technique and SB technique. DR = double-row, SB = suture-bridge, UCLA = University of California at Los Angeles.

In Figure 6, 6 included studies^[10–14,16]^ consisting of 541 patients (268 patients received SB treatment and 273 patients received DR treatment) investigated postoperative UCLA score. None heterogeneity among studies (*P* = .75, *I*^2^ = 0%) was found, so we used the fixed-effect model to pool the data. The overall estimate indicated that the pooled MD was −0.95 (95% CI = −1.70 to −0.20, *P* = .01), suggesting that SB and DR treatment had a statistically significant difference.

### 3.8 Comparison of ROM between SB and DR

**Figure 7:**
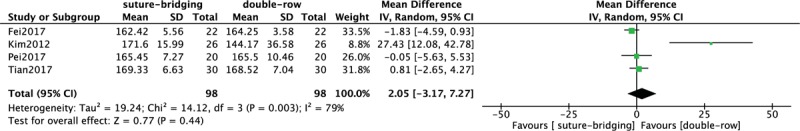
Forest plot of comparison: postoperative forward flexion between traditional DR technique and SB technique. DR = double-row, SB = suture-bridge.

**Figure 8:**
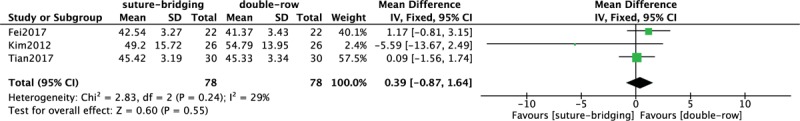
Forest plot of comparison: postoperative external rotation between traditional DR technique and SB technique. DR = double-row, SB = suture-bridge.

Four included studies^[11,13–15]^ including 98 SB surgery group cases and 98 DR surgery group cases provided the data in terms of postoperative forward flexion. A heterogeneity test revealed that a significant heterogeneity existed among the studies (*P* = .003, *I*^2^ = 79%) and the random-effect model was used. A pooled analysis revealed that there was no significant difference between SB surgery and DR surgery group (MD = 2.05, 95% CI = −3.17 to 7.27, *P* = .44) (Fig. 7). Comparison of postoperative external rotation between the 2 groups was conducted among 3 included studies,^[11,14,15]^ which contain 156 patients (78 patients received SB surgery and 78 patients received DR surgery treatment) in Figure 8. A low heterogeneity was found among studies (*P* = 0.24, *I*^2^ = 29%), so the fixed-effect model was used. The pooled result showed that the difference between SB surgery and DR surgery group was not statistically significant (MD = 0.39, 95% CI = −0.87 to 1.64, *P* = .55).

### 3.9 Comparison of re-tear rate between SB and DR

**Figure 9:**
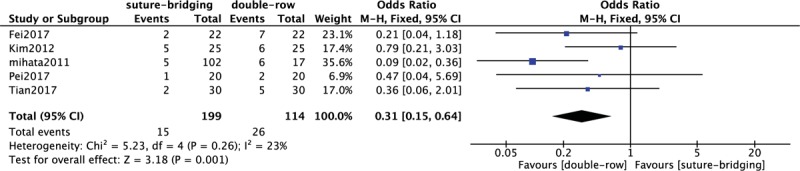
Forest plot of comparison: re-tear rate between traditional DR technique and SB technique. DR = double-row, SB = suture-bridge.

In Figure 9, 5 included studies^[10,11,13–15]^ consisting of 313 rotator cuff tear patients (199 patients received SB and 114 patients received DR technique) reported re-tear rate. A low heterogeneity among studies (*P* = .26, *I*^2^ = 23%) was found, so we used the fixed-effect model. The overall estimate indicated that the pooled OR was 0.31 (95% CI = 0.15–0.64, *P* = .001), suggesting that the difference was statistically significant, and the re-tear rate of DR intervention was higher than that of SB.

### 3.10 Sensitivity analysis and publication bias

**Figure 10:**
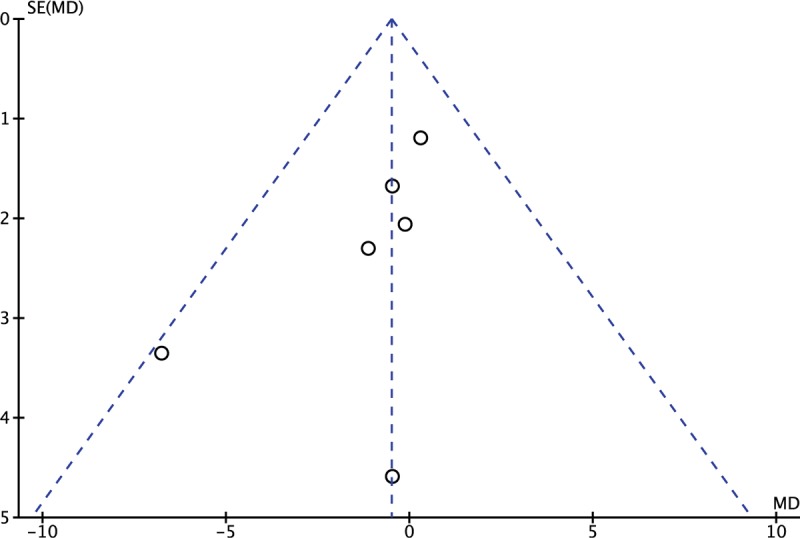
Funnel plot to test for publication bias. Each point represents a separate study for the indicated association. The vertical line represents the mean effects size. MD = mean difference, SE = standard error.

We performed a sensitivity analysis to assess the stability of the pooled results. Among the most studies, the heterogeneity results were not obviously altered after sequentially omitting each study, indicating that our results were statistically reliable. The funnel plot of the included studies is shown in Figure 10. The points in the funnel plot were almost symmetrically distributed, indicating that the publication bias was not apparent.

## 4 Discussion

### 4.1 Summary of main results

In this study, we identified 1 RCT and 6 observational studies for investigating the clinical outcomes of arthroscopic SB versus DR intervention. Our meta-analysis results showed that the differences were not statistically significant between the 2 interventions for ASES score, Constant score, VAS score, and ROM. However, a different result was discovered by UCLA score analysis. The difference of UCLA score was statistically significant between SB and DR intervention, and the SB technique proved it had a higher efficacy which is in accordance with some clinical results.^[15]^ In Mihata's study,^[10]^ for small or medium tears, the present study did not find a significant difference among the comparison in re-tear rates after arthroscopic rotator cuff repair. Therefore, any of the 2 arthroscopic repair techniques could be used to repair small or medium tears to obtain good structural and functional outcomes. On the other hand, a high re-tear rate was found after arthroscopic conventional DR repair for large or massive tears (41.7%), the additional suture bridges significantly decreased the re-tear rate for large and massive tears compared with the conventional DR techniques. The biomechanical study of Kim et al showed that footprint reconstruction of the rotator cuff using a DR repair not only can increase the area of tendon bone contact and reduce the gap formation and strain over the footprint when compared with a single-row repair, but also increase the ultimate failure load and provide maximal initial fixation strength.^[17–21]^ Meier et al also had proved that the initial strength and healing rates of DR suture are better than single-row and transosseous repairs, and the re-tear rate is less than single-row suture in the biomechanical comparison.^[22]^ However, compared with SB suture, there are many shortcomings in the DR suture. In laboratory studies, the mean pressurized contact area between the tendon and tuberosity insertion footprint with the SB technique was superior to that of the conventional DR technique. The SB technique has greater ultimate failure load and less gap formation than the DR technique.^[7,8]^ However, Barber et al showed triple-loaded suture anchors perform in a superior fashion to SB technology. Therefore, further studies are required to clarify the biomechanical advantage of the suture bridge.^[23]^ In addition, a DR rotator cuff repair, where each suture anchor is tied separately, is a technically demanding, time-consuming procedure.^[24]^ Compared to SB technique, DR technique had more knots to cause knot impingement and even re-tear. Hotta et al reported 9 cases (including single-row, DR and, suture reel method) in which, after rotator cuff repair surgery, osteolysis in the inferior surface of the acromion appeared to have been caused by impingement of the knots in the suture thread (knot impingement).^[25]^ Park et al showed that erosion in the acromion was observed in 2 of 118 patients (1.7%) in single-row groups and in 1 of 103 (1%) patients in SB groups, and there is no difference in acromial erosion in high-profile knots made by a single-row compared with SB repair with minimal knots.^[26]^ Rhee et al compared the repair integrity of arthroscopic rotator cuff repair between a knotless and a conventional knot-tying SB technique for patients with full-thickness rotator cuff tears, and the knotless group had a significantly lower re-tear rate compared with the conventional knot-tying group.^[27]^ Boyer et al also compared the functional and structural outcomes of tied and knot-less SB techniques for DR, suture-bridging cuff repair, and the knot-less tape-bridging construct showed a lower but not significant re-tear rate.^[28]^ To sum up, a knotless SB technique could be a new supplementary repair technique to conventional technique.

The re-tear rate in 7 included studies also should be discussed. On the whole, 15 (7.5%) re-tear under SB surgery was reported and 26 (22.8%) re-tear under DR surgery was reported in 7 included studies,^[10–16]^ which showed that SB treatment has the lower re-tear rate than DR treatment and is a better fixing technique. In Kim's study,^[11]^ 2 types of re-tear patterns were identified in the DR and SB group:

(1) unhealed tendons, 4 of 6 (66.6%) and 3 of 5 (60%), and
(2) medially ruptured tendons with a healed footprint, 2 of 6 (33.3%) and 2 of 5 (40%), respectively.

In Mihata's study,^[10]^ for the small to medium tears, no re-tear has been found at 6, 12, and 24 months after repair when the repaired tendon had been intact at 3 months. This result suggested that most of the small and medium tears may heal within 3 months after rotator cuff repair. In the large to massive tears, 3 of 13 (23.1%) re-tears were found without any traumatic episode at 6 months after repair. Therefore, careful examination may be necessary for at least 6 months after rotator cuff repair for the large or massive tears. Although the high rates of re-tears have been attributed to many factors, including the severity of the tear, tendon and bone quality, and muscle atrophy and fatty degeneration, repair techniques such as SB technique have been developed to improve the biomechanical properties of rotator cuff repair.^[29–32]^ However, Matthew's meta-analysis showed that DR and SB repairs were all secured with mattress sutures, and there were no differences in the rates of re-rupture between these methods for either size category. These findings suggest that suture technique may not affect re-rupture rates after rotator cuff repair.^[33]^ Hein et al also reported that Both DR and SB have lower re-tear rates in most tear size categories. No differences in re-tear rates were found between DR and SB at a minimum of 1 year of imaging follow-up.^[34]^ In addition, there were no surgical complications in 7 included studies,^[10–16]^ including neural injury, infection, or suture anchor problems. Stiffness of the joint is the most important complication after the operation, which is mainly related to the postoperative rehabilitation plan. Some researchers advocated early exercise to prevent joint stiffness, but some literatures indicated that early braking will be more conducive to the healing of the rotator cuff, and it does not increase the rate of long-term joint stiffness.^[35–37]^

The mean ± standard deviation (SD) operating time required for SB technique was 71.36 ± 12.59 minutes (range, 22–56 minutes), and the mean ± SD operating time for DR technique was 86.19 ± 18.84 minutes (range, 18–48 minutes) in Tian's study.^[14]^ The operating time required for SB technique was 92 minutes (range, 65–125 minutes), and the operating time for DR technique was 107 minutes (range, 75–145 minutes) in Pei's study.^[13]^ Therefore, the operating time for SB technique was less than that for DR technique, which partly can be attributed to the use of knotless extrusion screw.^[38]^ The number of anchors with SB technique was 1.4 ± 0.2 inner row anchor and 2.3 ± 0.3 outer row anchor, and the number of anchors with DR technique was 1.5 ± 0.3 inner row anchor and 1.5 ± 0.5 outer row anchor in Fei's study.^[15]^ In addition, 1.61 ± 0.48 inner row anchor and 2.16 ± 0.37 outer row anchor with SB technique, and 1.59 ± 0.51 inner row anchor and 1.63 ± 0.49 outer row anchor with DR technique in Tian's study^[14]^ were reported, which indicated that SB technique resulted in relatively large costs of inpatients.

### 4.2 Strengths and limitations of the study

To our knowledge, this is the first systematic review and meta-analysis with moderate quality RCTs and observational studies to compare the efficacy, safety and cost of arthroscopic SB and DR interventions for rotator cuff tear patients. In meta-analyses, adding more information from observational studies may aid in clinical reasoning and establish a more solid foundation for causal inferences.^[39]^ Some limitations of this study should be noted. First, the small sample size might have affected the significant difference between the 2 surgical procedures. Second, significant statistical heterogeneity of ASES score, VAS score, and ROM still existed among the included trials, which may be explained by the clinical diversity among trials. Third, our study ignored the diversity of used diagnostic criteria and etiology of the disease, and further research is needed to discover whether these conclusions apply to patients with varying degrees of rotator cuff tears. Last but not least, the included studies were mostly observational studies and not RCTs, and they largely relied on retrospectively collected data, resulting in a high risk of selection bias. More large-sample, multi-center, high-quality, RCTs are needed to verify the outcomes of this meta-analysis.

## 5 Conclusions

In conclusion, both arthroscopic SB and DR interventions have benefits in rotator cuff tear. SB treatment is more effective in UCLA score and has lower re-tear rate than DR treatment, which indicates that the arthroscopic SB technique could be recommended as the optical choice for rotator cuff tear. In view of the heterogeneity and different follow-up time, whether these conclusions are applicable should be further determined in future studies.

## Author contributions

**Conceptualization:** Tao Yang.

**Data curation:** Yi-Ming Ren, Yuan-Hui Duan, Yun-Bo Sun.

**Formal analysis:** Yi-Ming Ren, Hong-Bin Zhang, Tao Yang.

**Funding acquisition:** Meng-Qiang Tian.

**Investigation:** Tao Yang.

**Methodology:** Yi-Ming Ren.

**Project administration:** Hong-Bin Zhang.

**Software:** Yi-Ming Ren, Tao Yang.

**Supervision:** Hong-Bin Zhang, Yuan-Hui Duan, Yun-Bo Sun, Meng-Qiang Tian.

**Validation:** Yuan-Hui Duan, Yun-Bo Sun.

**Visualization:** Yi-Ming Ren, Hong-Bin Zhang.

**Writing – original draft:** Yi-Ming Ren, Hong-Bin Zhang.

**Writing – review and editing:** Yi-Ming Ren, Hong-Bin Zhang, Meng-Qiang Tian.
